# LncRNA HOTAIR facilitates proliferation and represses apoptosis of retinoblastoma cells through the miR-20b-5p/RRM2/PI3K/AKT axis

**DOI:** 10.1186/s13023-022-02206-y

**Published:** 2022-03-05

**Authors:** Ke Fu, Ke Zhang, Xiaoyu Zhang

**Affiliations:** 1 Department of Ophthalmology, The First Affiliated Hospital of Nanyang Medical College, No. 46 Chezhan South Road, Wolong District, Nanyang, 450000 Henan China; 2 Department of Ophthalmology, The First Affiliated Hospital of Zhengzhou University, Zhengzhou, 450000 Henan China; 3 Department of Ophthalmology, Henan Provincial People’s Hospital, The First Hospital Directly Under Henan Province, Zhengzhou, 450000 Henan China

**Keywords:** Retinoblastoma, Proliferation, Apoptosis, LncRNA HOTAIR, microRNA-20b-5p, RRM2, PI3K/AKT pathway, Xenograft tumor

## Abstract

**Purpose:**

Retinoblastoma (RB) represents an adolescent eye malignancy. Long non-coding RNA (LncRNA) HOTAIR shows aberrant expression in many malignancies. This research investigated the mechanism of HOTAIR in RB.

**Methods:**

Normal retinal cell lines (ARPE-19 and RPE-1) and RB cell lines (ORB50, Y79, HXO-RB44, and WERI-RB) were selected for detection of HOTAIR expression by qRT-PCR. sh-HOTAIR was delivered into Y79 and HXO-RB44 cells. Cell-cycle distribution, proliferation, and apoptosis were detected by CCK-8 assay and flow cytometry. Binding relationships among HOTAIR, miR-20b-5p, and RRM2 were confirmed using dual-luciferase assay. Roles of miR-20b-5p and RRM2 in RB cell-cycle distribution, proliferation, and apoptosis were ascertained by functional rescue experiments. Murine model of xenograft tumor was established, followed by detection of tumor growth and counting of Ki67-positive cells. Expressions of proliferation- and apoptosis-associated proteins and PI3K/AKT pathway-related proteins were determined by Western blot.

**Results:**

HOTAIR was elevated in RB cells relative to that in normal retinal cells and showed relatively high expression in Y79 and HXO-RB44 cells. sh-HOTAIR induced RB cell-cycle arrest, restrained proliferation, and strengthened apoptosis. HOTAIR functioned as the ceRNA of miR-20b-5p and targeted RRM2. RB cells had poorly-expressed miR-20b-5p and highly-expressed RRM2. miR-20b-5p downregulation or RRM2 overexpression facilitated RB cell-cycle and proliferation, suppressed apoptosis, and reversed the protective effect of sh-HOTAIR on RB. sh-HOTAIR reduced tumor growth and Ki67-positive cells in vivo and inactivated PI3K/AKT pathway.

**Conclusion:**

LncRNA HOTAIR upregulated RRM2 by competitively binding to miR-20b-5p and activated PI3K/AKT pathway, thereby facilitating proliferation and repressing apoptosis of RB cells.

## Introduction

Retinoblastoma (RB) constitutes the most universal intraocular malignancy commonly resulting from the RB tumor suppressor gene (Rb1) inactivation [1], with the characteristic manifestations of vision decline, red and irritated eyes, and leukocoria [2]. At present, roughly 8000 new cases are estimated each year, which requires ever-increasing medical costs, especially in countries with high fertility rates such as Asia and Africa [3]. If not treated in time, children with RB are at multiple life risks including RB cell invasion, malignant transformation of intracranial neuroblastoma, and heterochronogenous tumor [4]. Therefore, it’s of significant importance to explore the molecular mechanism underlying RB occurrence and development, thus opening up a novel therapeutic pathway for RB.

Long non-coding RNAs (LncRNAs), a set of non-coding RNA transcripts with exceeding 200 nucleotides act as oncogenes or tumor suppressors by dominating tumor-associated genes or pathways [5]. LncRNAs partake in gene regulation during the initiation and progression of RB [6, 7]. Microarray analysis has clarified elevated expression of lncRNA HOX transcript antisense RNA (HOTAIR) in RB tissues, contributing to the accelerated RB progression and metastasis [7]. Dong et al. have revealed the role of HOTAIR as a promising therapeutic target for RB, and knockdown of HOTAIR represses RB cell proliferative and invasive abilities [8]. Mechanically, lncRNAs can serve as competing endogenous RNAs (ceRNAs) and then bind to microRNA (miRNA) sites, thus modulating the expressions of mRNAs and target genes [9]. miRNAs are small noncoding RNAs with the potentials to modulate the expressions of protein-coding genes at the post-transcriptional level [10], which are implicated in critical physiological processes and pathological conditions such as carcinogenesis [11]. Yang et al. have suggested that HOTAIR modulates RB cell viability, apoptosis, and epithelial-mesenchymal transition via sponging miR-613 [12]. Nonetheless, the other potential ceRNA mechanism of HOTAIR in RB remains pending.

The downstream target miRNAs of HOTAIR are screened out using the bioinformatics method, among which miR-20b-5p can form a ceRNA network with HOTAIR in gastric cancer [13]. Emerging evidence has found aberrant expression of miR-20b-5p in human cancers and anti-tumor or oncogenic effects of miR-20b-5p on different malignancies in a context-dependent manner [14–16]. Importantly, overexpression of miR-20b-5p is proved to repress proliferation and facilitate apoptosis of RB cells [17]. However, whether HOTAIR can function as the ceRNA of miR-20b-5p to manipulate RB progression has not been elucidated before. This study thereupon probed into the ceRNA mechanism of lncRNA HOTAIR in RB, hoping to convey reference value for the improvement of diagnosis and treatment of RB.

## Materials and methods

### Ethics statement

The approval of the Ethics Committee of the First Affiliated Hospital of Nanyang Medical College was obtained before conducting this study. All experiments were implemented based on the ethical guidelines for the investigation of experimental pain in conscious animals.

### Cell lines and culture

Two normal human retinal epithelial cell lines (ARPE-19 and RPE-1) and 4 RB cell lines (SORb50, Y79, HXO-RB44, and WERI-Rb-1) were procured from ATCC (Manassas, VA, USA) and cultured at 37 °C with 5% CO_2_ in RPMI-1640 medium (Invitrogen, Carlsbad, CA, USA) supplemented with 10% fetal bovine serum (Life Technologies, Pleasanton, CA, USA) and 100 × penicillin–streptomycin (FT20016yy, Shanghai FanTai Biotechnology Co., Ltd., Shanghai, China). Mycoplasma contamination was stringently prevented and maintained under control during cell culture and subsequent cell treatment, and mycoplasma contamination was detected at regular intervals. Y79/HXO-RB44 cells were classified into 11 groups: blank group, sh-HOTAIR group (cells were transfected with lncRNA HOTAIR shRNA), sh-NC group (cells were transfected with negative control of shRNA), miR-inhi group (cells were transfected with miR-20b-5p inhibitor), miR-NC group (cells were transfected with the negative control of miR-20b-5p inhibitor), sh-HOTAIR + miR-inhi group (cells were transfected with lncRNA HOTAIR shRNA and miR-20b-5p inhibitor), sh-HOTAIR + miR-NC groups (cells were transfected with lncRNA HOTAIR shRNA and inhibitor NC), oe-RRM2 group (cells were transfected with RRM2 overexpression plasmid), oe-NC group (cells were transfected with empty plasmid), sh-HOTAIR + oe-RRM2 group (cells were transfected with lncRNA HOTAIR shRNA and RRM2 plasmid), and sh-HOTAIR + oe-NC group (cells were transfected with lncRNA HOTAIR shRNA and empty plasmid). All transfection plasmids were provided by Gene Pharma (Shanghai, China). The transfection process strictly complied with the instructions of Lipofectamine™ 2000 (Invitrogen), and the final concentration of each plasmid was 50 nmol/L. The subsequent experimentations were conducted 48 h later.

### Cell counting kit-8 (CCK-8) assay

After seeding in 96-well plates (4 × 10^3^ cells/100 μL), the transfected cells were added with 20 μL CCK-8 solutions in each well. After incubation for 4 h, the supernatant was abandoned, and each well was supplemented with 150 µL dimethyl sulfoxide, followed by 10-min gentle shaking. Subsequently, an enzyme-labeling measuring instrument (BIO-TEK, VT, USA) was employed to measure the optical density (OD) at 490 nm of each well. Finally, the cell viability curves were drawn, with X-axis symbolizing time and Y-axis symbolizing OD value.

### Apoptosis assay

Flow cytometry was adopted to analyze the cell cycle and cell apoptosis. A cell cycle detection kit (Roche, Indianapolis, IN, USA) was utilized to detect the cell cycle. In brief, 1 × 10^5^ Y79 and HXO-RB44 cells were added with 1 mL DNA staining solution, mixed, and subsequently supplemented with 10 μL propidium iodide (PI) staining solution, followed by mixture and 30 min-standing at room temperature in strict conformity with the instructions of the kits. Then, the cells were placed in a FACScan (Beckman Coulter, Fullerton, CA, USA) for analysis. The FlowJo software (Tree Star, San Carlos, CA, USA) was utilized to analyze the cell cycle. Cell apoptosis was measured by Annexin V-FITC and PI double staining. Briefly, 1 × 10^5^ Y79 and HXO-RB44 cells were rinsed and stained with Annexin V-FITC and PI using the FITC Annexin V apoptosis detection kit (BD Biosciences, Franklin Lakes, NJ, USA). Next, the cells were suspended in the binding buffer and then placed in a FACS Calibur flow cytometer (Beckman Coulter) for flow cytometry analysis. The number of apoptotic cells was quantified using FlowJo software (Tree Star).

### Quantitative real-time polymerase chain reaction (qRT-PCR)

Total RNA was extracted from RB cells or tissue samples using the TRIzol reagent (Thermo Fisher Scientific, Waltham, MA, USA). Agilent 2100 Bioanalyser, RNA 6000 LabChip kit, and Agilent 2100 Expert software (Agilent Technologies, CA, USA) were utilized to analyze RNA integrity. RNA was reversely transcribed into cDNA using the Transcript First Strand cDNA Synthesis kit (Roche, Germany) and random hexamer primers. qRT-PCR was carried out using FastStart Universal SYBR Green Master (Roche) on a Bio-Rad C1000 Thermal Cycler (Bio-Rad, CA, USA), with GAPDH acting as the internal control. The design and synthesis of primers were completed by Guangzhou Boxin Biotechnology Co., Ltd (Guangzhou, China) (Table 1).

**Table 1:** Primer sequence for qRT-PCR

Gene	Sequence (5′–3′)
miR-20b-5p	F: CCTAGTAGTGCCAAAGTGCT
	R: CCAGGAGTACTA GAAGTGATCA
U6	F: CTCGCTTCGGCAGCACA
	R: GTGTCGTGGAGTCGGCAA
HOTAIR	F: GGTCTTGCCTCCTCTCTGTG
	R: CTCTGGCCAGGAAAAGAGTG
GAPDH	F: ACCAGGTATCTGCTG GTTG
	R: TAACCATGATGTCAGCGTGGT

### Western blot analysis

The protein concentration was detected by bicinchoninic acid assay. After mixing with loading buffer, the proteins were denatured by boiling. Next, the proteins were separated by electrophoresis and transferred into polyvinylidene difluoride membranes. Subsequently, the membranes were incubated with the primary antibodies and then the secondary antibody goat anti-mouse anti-IgG H&L (HRP) (1/2000, ab205719, Abcam). Then, the membranes were developed using the enhanced chemiluminescence. The levels of Ki67 (1/1000, ab16667, Abcam, Cambridge, MA, USA), Cleaved Caspase-3 (20 µg/mL, ab32042, Abcam), Bcl-2 (1/500, ab692, Abcam), RRM2 (1 µg/mL, ab57653, Abcam, Cambridge, MA, USA), AKT (1/500, ab8805, Abcam), phosphorylated AKT (p-AKT, 1/500, ab38449, Abcam), phosphatidylinositol 3-kinase (Pl3K) (1/500, ab154598, Abcam), p-Pl3K (1/500, ab182651, Abcam) were determined, with GAPDH (1/500, ab8245, Abcam) acting as the internal reference. The quantification of band intensity was processed using Image-Pro Plus 6.0 software (Olympus, Tokyo, Japan).

### Dual-luciferase reporter gene assay

The reporter plasmid HOTAIR-WT/RRM2-WT was prepared by inserting the pmirGLO vector (Promega, Madison, WI, USA) with HOTAIR sequences containing the target site of miR-20b-5p. The negative control for reporter plasmid, named HOTAIR-MUT/RRM2-MUT was set by mutating and inserting the target site. As aforementioned [18], 500 µL HEK-293 cells (ATCC) were seeded in 24-well plates (2 × 10^4^) and co-transfected with the constructed vectors (50 ng/µL) and miR-20b-5p mimic (20 µM) using Lipofectamine TM 2000 Reagent (Invitrogen) upon 80% confluence for 48 h. Luciferase activity assay was performed via dual-luciferase reporter assay system (Promega) to detect the relative activities of firefly luciferase and ranilla luciferase.

### Bioinformatics analysis

Downstream miRNAs of lncRNA HOTAIR were predicted through the databases ENCORI (http://starbase.sysu.edu.cn/agoClipRNA.php?source=lncRNA) and LncBase (http://carolina.imis.athena-innovation.gr/diana_tools/web/index.php?r=lncbasev2%2Findex-predicted) and intersections were taken to identify the target miRNA. The downstream targets of the target miRNA were predicted through databases ENCORI (http://starbase.sysu.edu.cn/), RNAInter (http://www.rna-society.org/rnainter/), Targetscan (http://www.targetscan.org/vert_71/), and miRDB (http://www.mirdb.org/). The coexpression relationship of genes was searched through the Coexpedia database (http://www.coexpedia.org/), and scored to further screen the target genes.

### Xenograft study

BALB/c mice between the age of 4 to 6 weeks (weighing 18 to 25 g) were supplied by the Laboratory Animal Center of Huazhong University of Science and Technology [SYXK (Hubei) 2010-0057, Wuhan, Hubei, China]. Briefly, after transfecting with HOTAIR shRNA or sh-NC, Y79 cells were incubated and washed with PBS. Mice in each group were subcutaneously injected with 5 × 10^6^ cells into one side of the posterior flank. The tumor length (L) and width (W) were measured with a caliper to calculate the tumor volume [V = (L × W^2^)/2]. Mice were intraperitoneally administered with 800 mg/kg pentobarbital sodium for euthanasia 28 days later. Subsequently, the tumors were removed and tumor weights were measured. Tumor tissues were paraffin-embedded and subjected to immunohistochemical (IHC) staining. Altogether 24 BALB/c mice were used for xenograft study and allocated to the sh-NC group (mice injected with Y79 cells stably transfected with sh-NC) and sh-HOTAIR group (mice injected with Y79 cells stably transfected with HOTAIR shRNA), N = 12. Among them, 6 mice were used for observation and measurement of the tumor growth rate, volume, and weight after extracting tumor tissues 28 days later for the detection of Cleaved Caspase-3 and Bcl-2 levels by Western blot, while the remaining 6 mice were used for detection of the expression of Ki67 using IHC assays.

#### IHC assays

The tumors from nude mice were fixed, embedded, and sectioned (5 μm). IHC staining was performed as instructed by the kit (Abcam). After overnight incubation with the primary antibody Ki67 (1:200, ab245113, Abcam), the tumor sections were incubated with the secondary antibody goat anti-mouse IgG H&L (Alexa Fluor® 488) (ab150113, Abcam) for 30 min. This step was followed by the addition of 3,3-diaminobenzidine H_2_O_2_ solution (Zhongshan Golden Bridge, Beijing, China) for development, and counterstaining with hematoxylin. Five images of the regions of interest without overlapping were taken and the positive cells per image were quantified using Image-Pro Plus 6.0 software. The results were presented as the average percentage of positive cells per image. The group without the primary antibody served as the negative control group.

### Statistical analysis

Data were processed using the SPSS 18.0 software (IBM Corp. Armonk, NY, USA) and GraphPad Prism 8.01 (GraphPad Software, San Diego, CA, USA). Measurement data are presented in the form of mean ± standard deviation. The *t* test was adopted for pairwise comparisons. The one-way analysis of variance was adopted for multigroup comparisons, followed by Tukey's multiple comparisons test was used for the post hoc test. The *p* < 0.05 was suggestive of significant differences.

## Results

### Downregulation of HOTAIR repressed RB cell proliferation and accelerated apoptosis

**Fig. 1:**
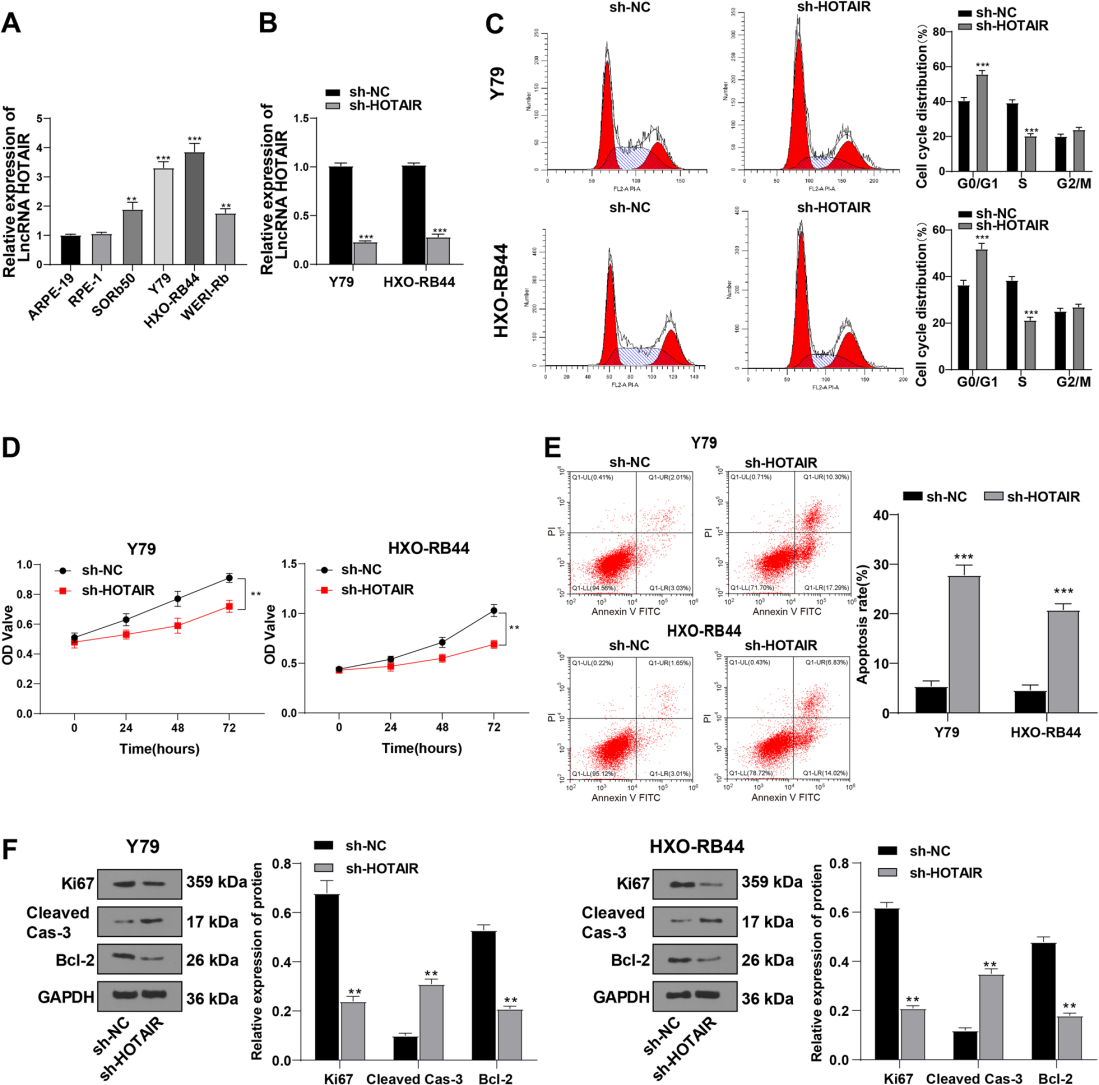
Downregulation of HOTAIR repressed RB cell proliferation and promoted apoptosis. Two types of normal retinal cells (ARPE-19 and RPE-1) and 4 types of RB cell lines (ORB50, Y79, HXO-RB44, and WERI-RB) were used. **A** HOTAIR expression was determined by qRT-PCR. After introducing sh-HOTAIR into Y79 and HXO-RB44 cells, **B** HOTAIR expression was determined by qRT-PCR. **C** Cell cycle was examined by flow cytometry; **D** cell proliferative ability was assessed by CCK-8 assay. **E** Cell apoptosis was assessed by flow cytometry. **F** Expressions of Ki67, Cleaved Caspase-3, and Bcl-2 were determined by Western blot. Cell experiment was repeated 3 times independently. The results were presented as mean ± standard deviation. Data in panel **A** were analyzed using the one-way ANOVA, and data in panels **B**–**F** were analyzed using the *t* test, followed by Tukey’s multiple comparisons test. In panel **A**, versus ARPE-19 or RPE-1; in panels **B**–**F**, versus corresponding control groups. **p < 0.01, ***p < 0.001. The relative expression of HOTAIR was normalized to that in ARPE-19 cells, with GAPDH as the internal reference; the relative expressions of Ki67, Cleaved Caspase-3, and Bcl-2 were normalized to the internal reference GAPDH

HOTAIR is highly-expressed in the tumor tissues of RB, which serves as a predictor of poor prognosis in patients with RB [12], implying a possible role of HOTAIR in the initiation and development of RB. qRT-PCR was carried out to explore the differential expression patterns of HOTAIR in normal retinal cells (ARPE-19 and RPE-1) and RB cells (SORb50, Y79, HXO-RB44, and WERI-Rb), which demonstrated that normal retinal cells ARPE-19 and RPE-1 showed no significant difference in HOTAIR expression (ARPE-19 cells were used as the normal control in the subsequent experiments), while RB cell lines showed an upregulation of HOTAIR expression (all *p* < 0.01) (Fig. 1A). The Y79 and HXO-RB44 cells having relatively high expression of HOTAIR were used as study subjects in the following experimentation.

To further examine the effect of HOTAIR on RB occurrence and development, sh-HOTAIR was introduced into Y79 and HXO-RB44 cells to silence HOTAIR, and qRT-PCR results confirmed the transfection efficiency (all *p* < 0.001) (Fig. 1B). Flow cytometry demonstrated that downregulation of HOTAIR blocked the RB cells in the G0/G1 phase of the cell cycle, and the number of RB cells in the S phase was considerably decreased (all *p* < 0.05) (Fig. 1C). CCK-8 assay indicated that downregulation of HOTAIR exerted a limitation on cell proliferation (all *p* < 0.01) (Fig. 1D). Flow cytometry exhibited notably increased apoptosis rates after silencing HOTAIR (all *p* < 0.001) (Fig. 1E). Western blot showed decreased levels of Ki67 and Bcl-2 and increased levels of Cleaved Caspase-3 in the Y79 and HXO-RB44 cells after silencing HOTAIR (all *p* < 0.01) (Fig. 1F). Taken together, silencing HOTAIR impeded proliferation and accelerated apoptosis of RB cells.

### HOTAIR modulated RB cell proliferation and apoptosis by serving as the ceRNA of miR-20b-5p

**Fig. 2:**
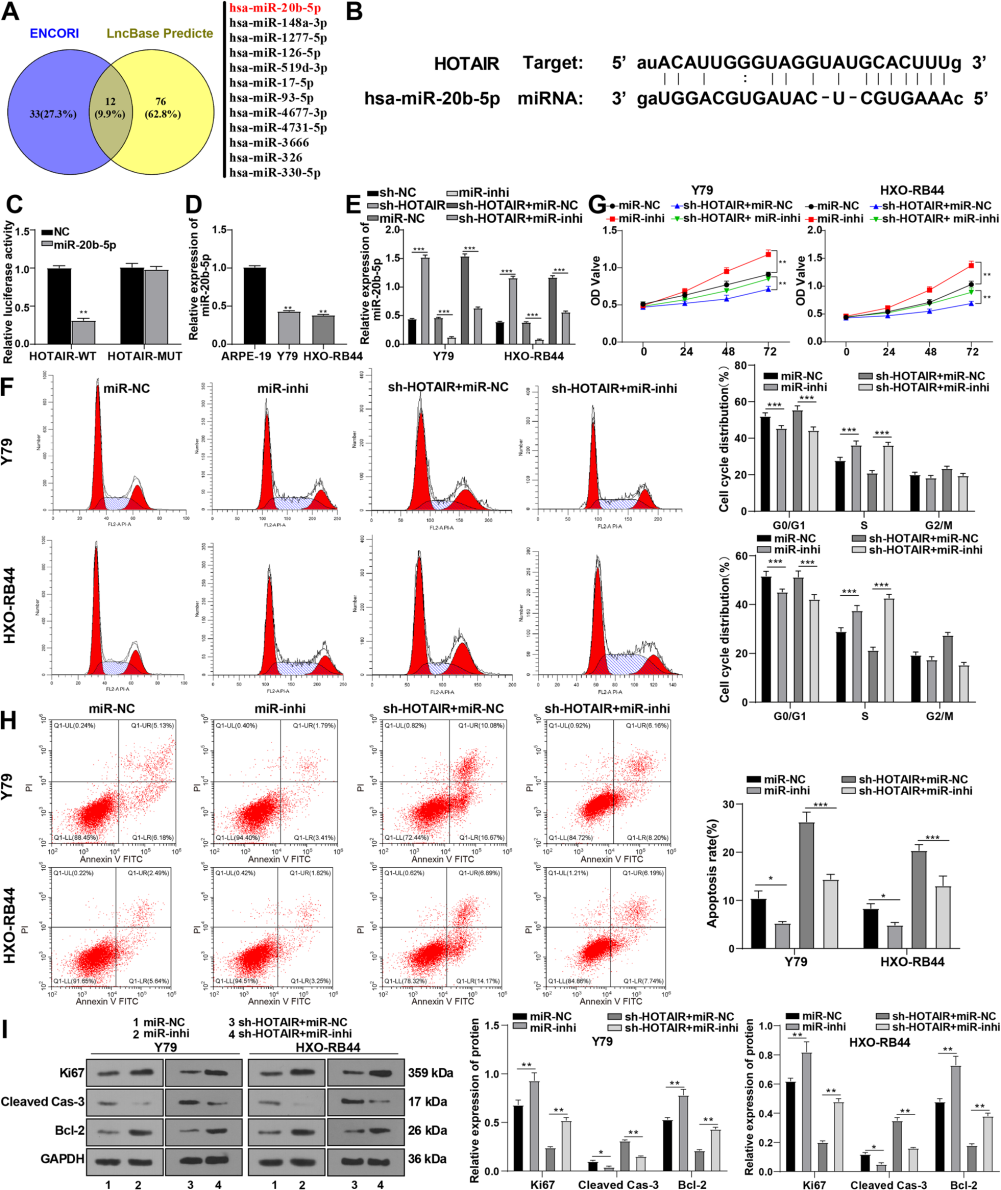
HOTAIR regulated RB cell proliferation and apoptosis by serving as the ceRNA of miR-20b-5p. **A** Downstream miRNAs of lncRNA HOTAIR were predicted on the ENCORI and LncBase databases. **B** The binding site of HOTAIR and miR-20b-5p was predicted on the ENCORI database. **C** The binding relationship between HOTAIR and miR-20b-5p was verified using dual-luciferase assay. **D** miR-20b-5p expression in normal retinal cells ARPE-19 and RB cells was determined by qRT-PCR. **E** miR-20b-5p expression in RB cells under different treatments was determined by qRT-PCR. The combined treatment of HOTAIR silencing and miR-20b-5p downregulation using miR-20b-5p inhibitor was implemented on Y79 and HXO-RB44 cells. **F** Cell cycle was examined by flow cytometry. **G** Cell proliferative ability was assessed by CCK-8 assay. **H** Cell apoptosis was assessed by flow cytometry. **I** Expressions of Ki67, Cleaved Caspase-3, and Bcl-2 were determined by Western blot. Cell experiment was repeated 3 times independently. Data were presented as mean ± standard deviation. Data comparisons among multiple groups were analyzed using the one-way ANOVA, followed by Tukey’s multiple comparisons test, and pairwise comparisons were analyzed using *t* test. *p < 0.05, **p < 0.01, ***p < 0.001. The relative expression of miR-20b-5p was normalized to that in ARPE-19 cells with U6 as the internal reference; the relative expressions of Ki67, Cleaved Caspase-3, and Bcl-2 were normalized to the internal reference GAPDH

As aforementioned, silencing HOTAIR inhibited the malignant biological behaviors of RB. Nevertheless, the downstream mechanism of HOTAIR remained elusive. LncRNA HOTAIR regulates RB cell proliferation and migration through the ceRNA mechanism [12]. To dig into the regulation mechanism of HOTAIR in RB cells, the downstream target miRNAs of HOTAIR were predicted through the ENCORI and LncBase databases, and 12 miRNAs were identified by taking the intersection (Fig. 2A). Among them, miR-20b-5p is the only one reported to repress RB cell proliferation and enhance apoptosis when overexpressed [17]. There is currently no report revealing the association between the remaining 11 miRNAs and RB development. We predicted the existence of a potential binding site between HOTAIR and miR-20b-5p on the ENCORI database (Fig. 2B), and the binding relationship between HOTAIR and miR-20b-5p was confirmed by dual-luciferase reporter gene assay (*p* < 0.01) (Fig. 2C). Therefore, we detected the expression of miR-20b-5p in normal retinal cells ARPE-19 and RB cells Y79 and HXO-RB44, and found that miR-20b-5p expression was significantly decreased in Y79 and HXO-RB44 cells compared with that in ARPE-19 cells (*p* < 0.01) (Fig. 2D). For this reason, we speculated that HOTAIR affected the malignant biological behaviors of RB by competitively binding to miR-20b-5p. By means of qRT-PCR, we detected miR-20b-5p expression in Y79 and HXO-RB44 cells after downregulation of HOTAIR and found that silencing HOTAIR notably increased miR-20b-5p expression (*p* < 0.001) (Fig. 2E). Overall, HOTAIR may affect RB cells by modulating miR-20b-5p expression.

To probe into the action of miR-20b-5p, we suppressed miR-20b-5p expression in RB cells (Y79 and HXO-RB44) via miR-20b-5p inhibitor, and discovered a decline in miR-20b-5p expression by qRT-PCR (all *p* < 0.001) (Fig. 2E). At the same time, the miR-inhi group exhibited promoted cell cycle progression, decreased number of G0/G1-phase cells, increased number of S-phase cells (all *p* < 0.01) (Fig. 2F), enhanced cell multiplication (all *p* < 0.01) (Fig. 2G), and suppressed cell apoptosis in comparison with the miR-NC group (all *p* < 0.01) (Fig. 2H). Furthermore, Ki67 expression and Bcl-2 protein expression were elevated and Cleaved Caspase-3 expression was decreased after downregulating miR-20b-5p (all *p* < 0.01) (Fig. 2I). Overall, downregulation of miR-20b-5p could stimulate RB cell cycle progression and multiplication and impede apoptosis.

A functional rescue experiment was conducted to figure out whether HOTAIR played a role in RB progression by affecting miR-20b-5p. The combined treatment of HOTAIR silencing and miR-20b-5p inhibitor was performed on Y79 and HXO-RB44 cells (sh-HOTAIR + miR-inhi group). Downregulation of miR-20b-5p was observed by qRT-qPCR after the combined treatment (all *p* < 0.001) (Fig. 2E). Compared with sh-HOTAIR treatment alone, the combined treatment of sh-HOTAIR + miR-inhi promoted cell cycle progression, showing concretely in the decreased number of cells in the G0/G1 phase and the increased number of cells in the S phase, facilitated cell proliferation and suppressed apoptosis (all *p* < 0.01) (Fig. 2F/H), upregulated Ki67 and Bcl-2 and downregulated Cleaved Caspase-3 in Y79 and HXO-RB44 cells (all *p* < 0.01) (Fig. 2I), indicating that weak expression of miR-20b-5p abrogated the inhibitory effect of sh-HOTAIR on RB. Taken together, HOTAIR regulated RB cell proliferation and apoptosis by functioning as the ceRNA of miR-20b-5p.

### miR-20b-5p targeted RRM2

**Fig. 3:**
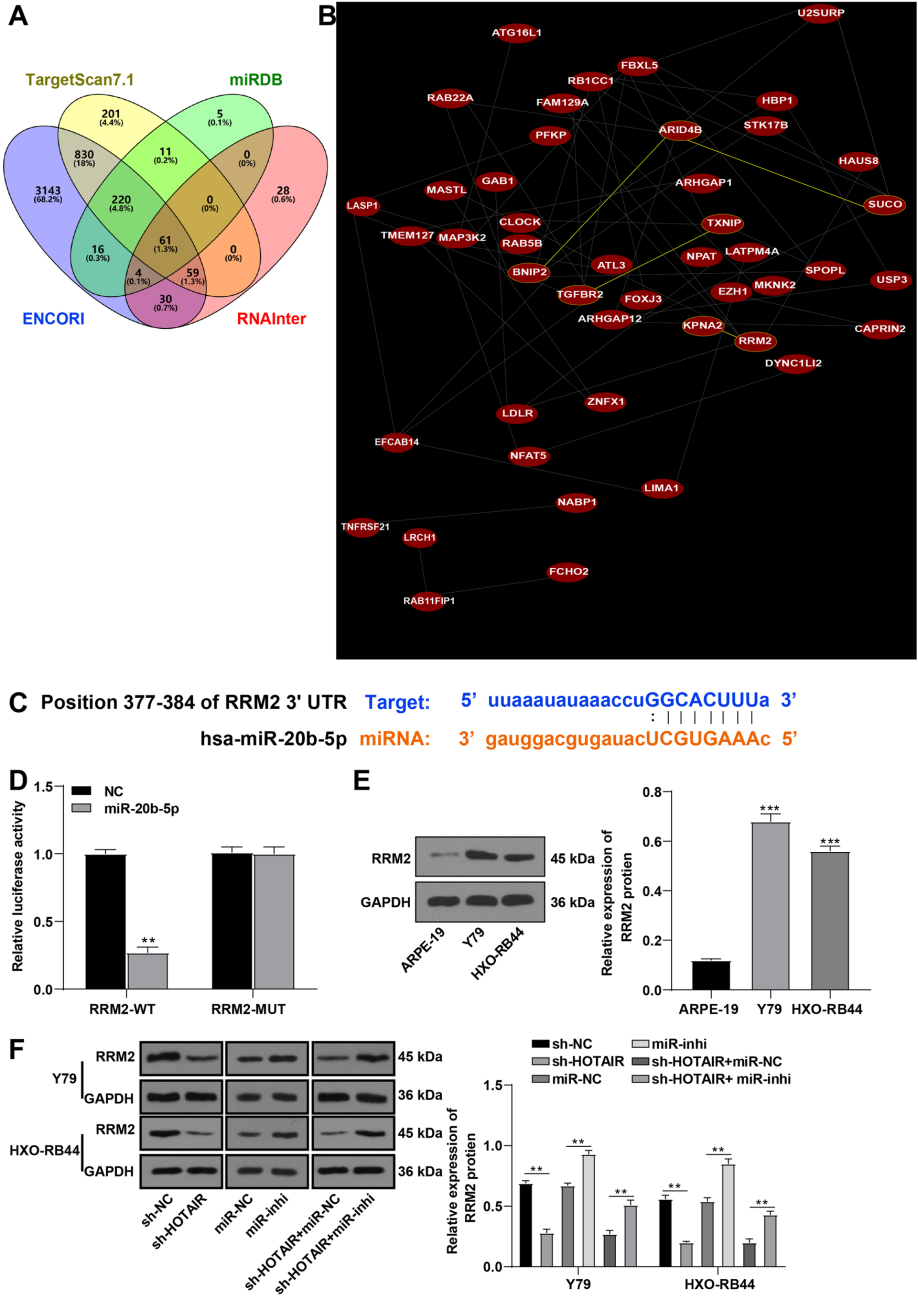
miR-20b-5p targeted RRM2. **A** Downstream target genes of miR-20b-5p were predicted on the ENCORI, RNAInter, Targetscan, and miRDB databases. **B** Coexpression relationship of target genes was searched on the Coexpedia database. **C** The binding site of miR-20b-5p and RRM2 was predicted on the ENCORI database. **D** The binding relationship between miR-20b-5p and RRM2 was verified using a dual-luciferase assay. **E** RRM2 level in normal retinal cells ARPE-19 and RB cells was detected using Western blot. **F** RRM2 level in RB cells Y79 and HXO-RB44 under different treatments was measured by Western blot. Cell experiment was repeated 3 times independently. The results were presented as mean ± standard deviation. Data in panels **E** and **F** were analyzed using the one-way ANOVA, and data in panel **D** were analyzed using the *t* test, followed by Tukey’s multiple comparisons test. **p < 0.01, ***p < 0.001. The relative expression of RRM2 was normalized to the internal reference GAPDH

To determine the downstream targets regulated by HOTAIR/miR-20b-5p, target genes of miR-20b-5p were predicted through StarBase, Targetscan, miRDB, and RNAInter databases (Fig. 3A). Then, we searched coexpression relationship of genes through the Coexpedia database to further screen the target genes. Based upon the provided coexpression scores, we screened two target genes with the highest scores: RRM2 and KPNA2 (score = 99.829, 92.738) (Fig. 3B). Overexpression of RRM2 exhibits the potential to stimulate tumor cell proliferation and migration [19–21]. Cell cycle is the most significantly upregulated pathway in RB tissues and RRM2 is identified as one of the critical genes in close relation to cell cycle regulation [22]. Thereby, we assumed that HOTAIR/miR-20b-5p might participate in RB by manipulating RRM2 expression. The binding site of miR-20b-5p and RRM2 3’UTR was predicted on the ENCORI database (Fig. 3C), and their binding relationship was confirmed by the dual-luciferase reporter assay (*p* < 0.01) (Fig. 3D). Western blot demonstrated that RRM2 expression was noticeably increased in Y79 and HXO-RB44 cells in comparison to that in ARPE-19 cells (*p* < 0.01) (Fig. 3E). Thereafter, we assumed that RRM2 might be implicated in RB occurrence and development through the regulation of cell cycle. After detecting RRM2 expression in Y79 and HXO-RB44 cells, we figured out that HOTAIR silencing significantly reduced RRM2 expression, whereas inhibition of miR-20b-5p (miR-inhi group or sh-HOTAIR + miR-inhi group) increased RRM2 expression (all *p* < 0.01) (Fig. 3F). Briefly, miR-20b-5p targeted RRM2.

### Overexpression of RRM2 attenuated the inhibition of sh-HOTAIR on RB cell proliferation

**Fig. 4:**
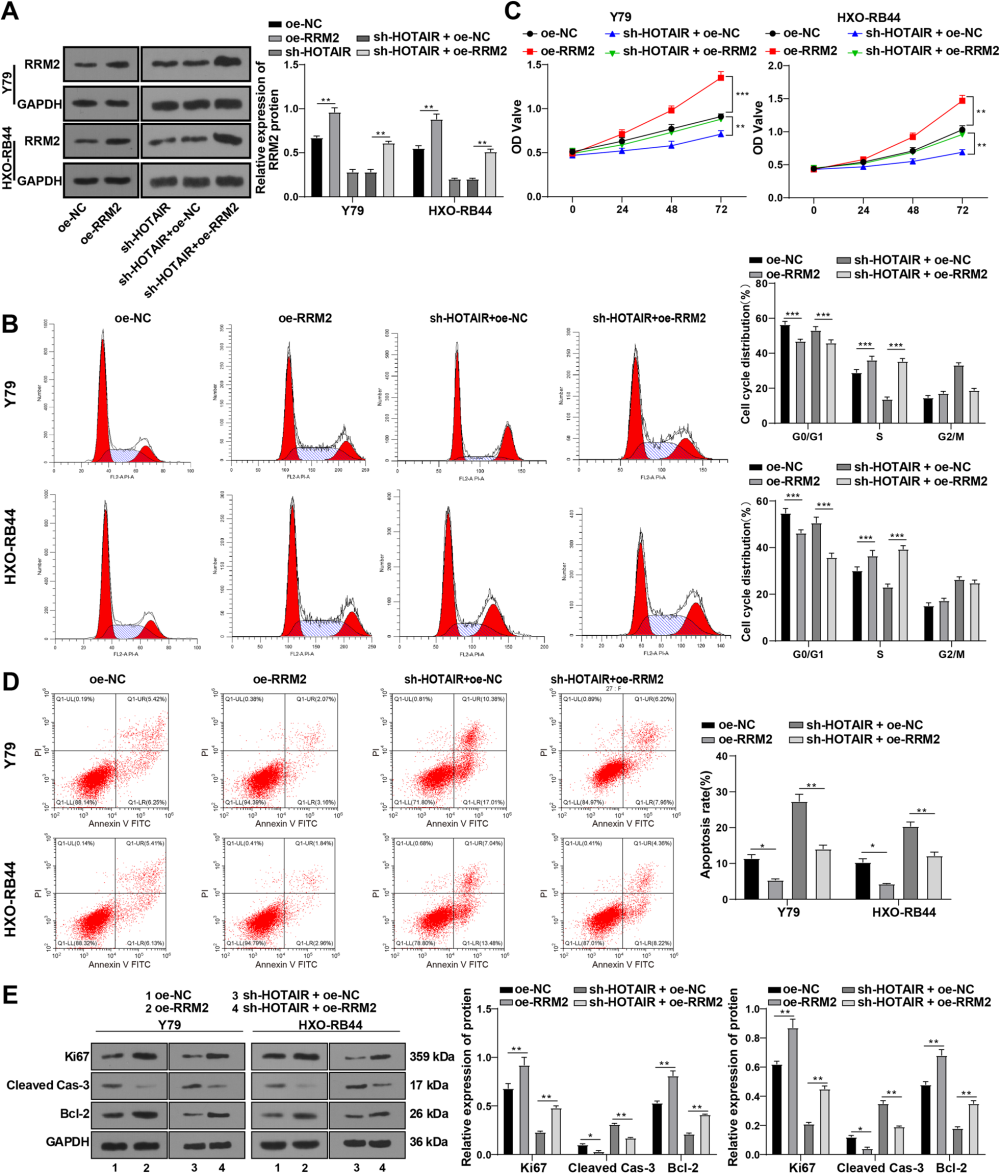
Overexpression of RRM2 reversed the inhibitory effect of sh-HOTAIR on RB cell proliferation. After treating Y79 and HXO-RB44 cells with the combination of sh-HOTAIR and oe-RRM2, **A** RRM2 level was measured by Western blot. **B** Cell cycle was examined by flow cytometry. **C** Cell proliferative ability was assessed by CCK-8 assay. **D** Cell apoptosis was assessed by flow cytometry. **E** Expressions of Ki67, Cleaved Caspase-3, and Bcl-2 were determined by Western blot. Cell experiment was repeated 3 times independently. The results were presented as mean ± standard deviation. Data in panel **A** were analyzed using the one-way ANOVA, and data in panels **B**–**E** were analyzed using the *t* test, followed by Tukey’s multiple comparisons test. *p < 0.05, **p < 0.01, ***p < 0.001. The relative expressions of all proteins were normalized to the internal reference GAPDH

We then further dug into the function of RRM2 in RB by delivering RRM2 overexpression plasmid into RB cells to upregulate RRM2, and found augmented RRM2 expression (all *p* < 0.001) (Fig. 4A). Through flow cytometry and CCK-8 assay, we found out that cell cycle progression was stimulated, G0/G1-phase cells decreased, S-phase cells increased (all *p* < 0.01) (Fig. 4B), cell proliferation was enhanced (all *p* < 0.01) (Fig. 4C), and cell apoptosis was remarkably repressed after RRM2 overexpression (all *p* < 0.01) (Fig. 4D). Moreover, the oe-RRM2 group displayed augmented expressions of Ki67 and Bcl-2 and diminished Cleaved Caspase-3 expression (all *p* < 0.01) (Fig. 4E). Altogether, RRM2 could accelerate RB cell cycle progression and proliferation and jeopardize RB cell apoptosis.

To ascertain that HOTAIR, miR-20b-5p, and RRM2 affected RB cell progression via the ceRNA mechanism, we transfected cells with sh-HOTAIR and oe-RRM2 (the sh-HOTAIR + oe-RRM2 group). Western blot revealed that in contrast to that in the sh-HOTAIR + oe-NC group, the RRM2 expression in the sh-HOTAIR + oe-RRM2 group was notably elevated (*p* < 0.01) (Fig. 4A). Then, the cell cycle and proliferative abilities of Y79 and HXO-RB44 cells were assessed by flow cytometry and CCK-8 assay. Compared to the sh-HOTAIR + oe-NC group, the sh-HOTAIR + oe-RRM2 group showed facilitated cell cycle progression with decreased number of cells in the G0/G1 phase and increased number of cells in the S phase, and enhanced proliferation (all *p* < 0.01) (Fig. 4B/C). Flow cytometry showed declined cell apoptosis in the sh-HOTAIR + oe-RRM2 group. Furthermore, the sh-HOTAIR + oe-RRM2 group displayed increased levels of Ki67 and Bcl-2 and decreased levels of Cleaved Caspase-3 (all *p* < 0.01) (Fig. 4D/E). Altogether, RRM2 overexpression reduced the suppressive action of sh-HOTAIR on RB cell proliferation.

### HOTAIR silencing repressed PI3K/AKT pathway activation via the miR-20b-5p/RRM2 axis

**Fig. 5:**
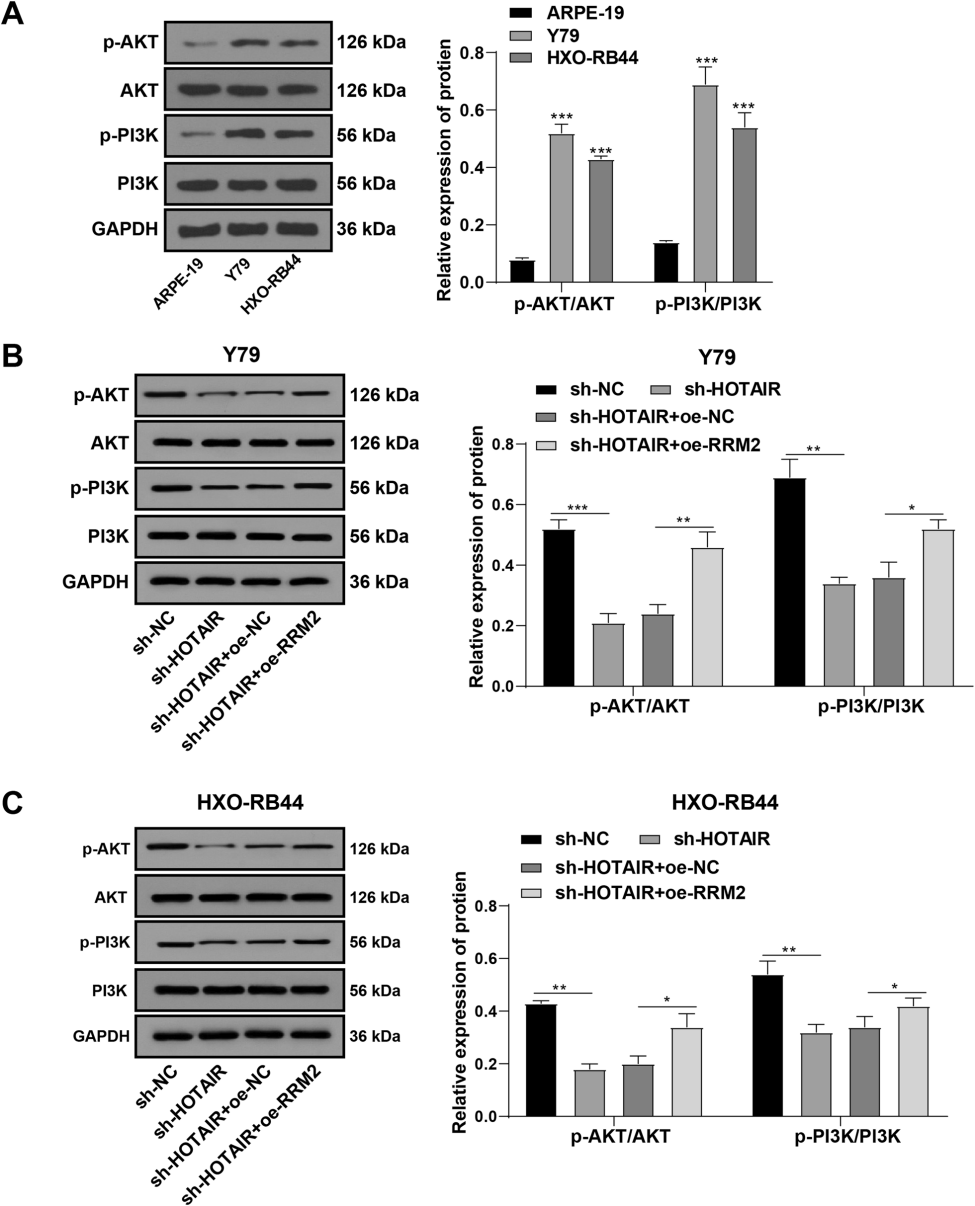
HOTAIR silencing repressed the activation of PI3K/AKT pathway via the miR-20b-5p/RRM2 axis. The levels of PI3K/AKT pathway-related proteins in normal retinal cell line ARPE-19 (**A**) and RB cell lines Y79 (**B**) and HXO-RB44 (**C**) were determined by Western blot. Cell experiment was repeated 3 times independently. The results were described as mean ± standard deviation and analyzed using the one-way ANOVA, followed by Tukey’s multiple comparisons test. In panel **A**, versus ARPE-19; in panels **B**/**C**, versus corresponding control groups. *p < 0.05, **p < 0.01, ***p < 0.001. The relative expressions of all proteins were normalized with GAPDH as the internal reference

The PI3K/AKT pathway exerts a regulatory function on RB cell proliferation and apoptosis [23]. RRM2 engages in the modulation of cancer cell proliferation, migration, and invasion via the PI3K/AKT pathway [21, 24]. The results above confirmed the ceRNA network of HOTAIR, miR-20b-5p, and RRM2. To further identify the downstream signaling pathway regulated by the HOTAIR/miR-20b-5p/RRM2 axis, we detected expressions of the PI3K/AKT pathway-related proteins in normal retinal cells ARPE-19 and RB cells Y79 and HXO-RB44 by Western blot, which manifested increased expressions of p-AKT/AKT and p-PI3K/PI3K in Y79 and HXO-RB44 cells and activation of the PI3K/AKT pathway (*p* < 0.05) (Fig. 5A). Silencing HOTAIR reduced the ratio of p-AKT/AKT and p-PI3K/Pl3K, whereas overexpression of RRM2 partially nullified the inhibitory action of sh-HOTAIR on the PI3K/AKT pathway (Fig. 5B/C). In short, silencing HOTAIR jeopardized the activation of the PI3K/AKT pathway via the miR-20b-5p/RRM2 axis.

### HOTAIR silencing restrained RB growth in vivo

**Fig. 6:**
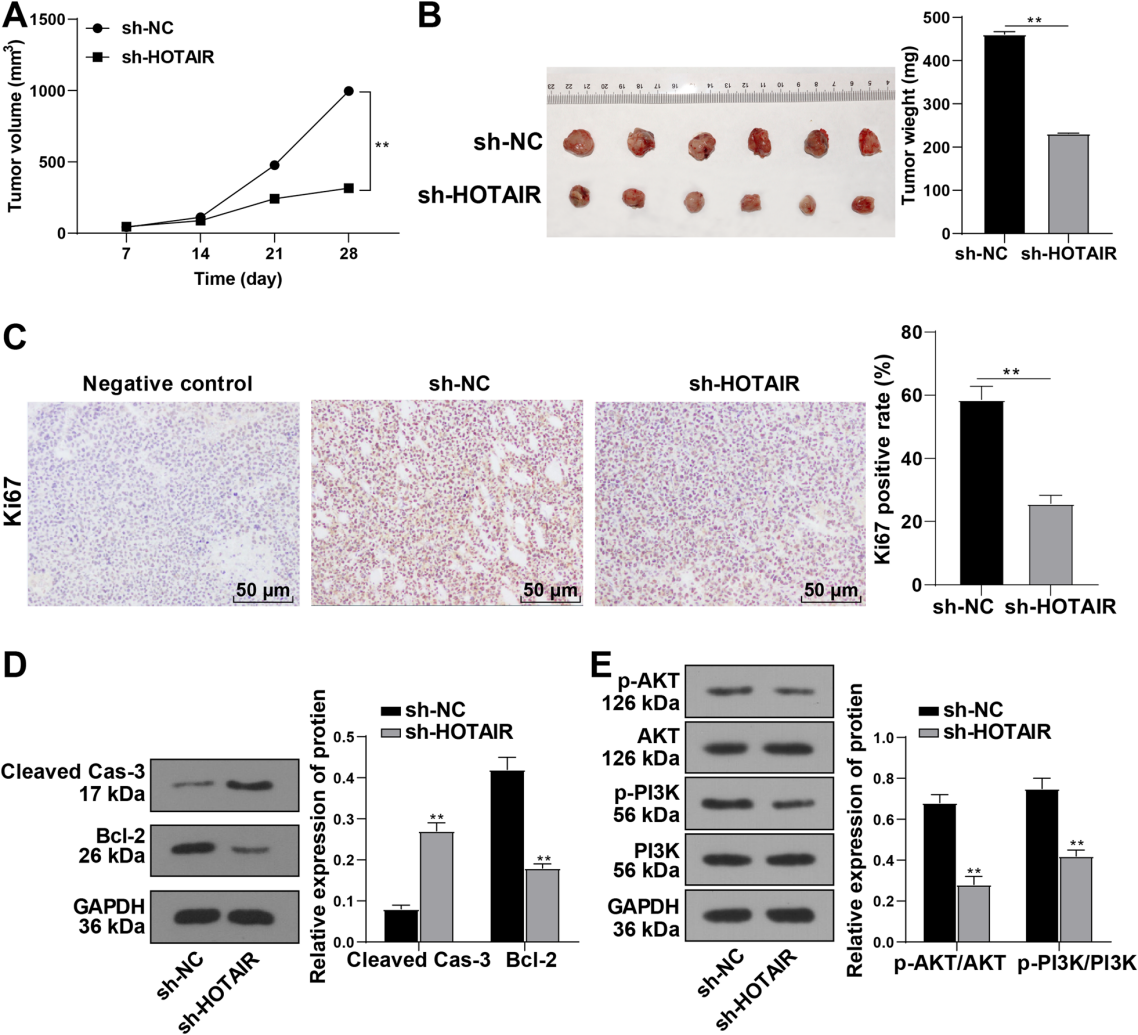
HOTAIR silencing restrained RB growth in vivo. **A** Tumor volume was calculated by measuring length and width every 7 days after injection. **B** After 28 days, tumors were extracted from nude mice, and tumor weight was measured. **C** Ki67-positive cells were counted by IHC assay (the scale bar denotes 100 μm). **D**–**E** Expressions of Cleaved Caspase-3, Bcl-2, and phosphorylation levels of the PI3K/AKT pathway were determined by Western blot. N = 6. The results were presented as mean ± standard deviation and analyzed using the *t* test. **p < 0.01. The relative expressions of all proteins were normalized with GAPDH as the internal reference

In animal experiments, Y79 cells stably transfected with HOTAIR shRNA or sh-NC were injected into nude mice to confirm the effect of HOTAIR on the tumorigenesis of RB cells in vivo. Consistent with the results obtained by in vitro assay, the tumor growth rate was decreased notably after silencing HOTAIR in vivo (Fig. 6A). The nude mice were euthanized after 28 days, and the tumors were taken out and weighed. The tumor weight and the rate of Ki67-positive cells of mice treated with sh-HOTAIR were dramatically lowered compared to those of the mice in the control group (Fig. 6B, C) (all *p* < 0.01). The expression of Cleaved Caspase-3 was augmented while the expression of Bcl-2 was diminished in the tumor tissues after silencing HOTAIR (Fig. 6D). Additionally, the phosphorylation levels of PI3K and AKT were considerably declined, revealing consistent results to the in vitro assay (all *p* < 0.01) (Fig. 6E). Collectively, HOTAIR silencing could suppress the growth of RB tumors by impeding the activation of the PI3K/AKT pathway in nude mice.

## Discussion

RB may evolve into a sporadic or hereditary form and even pose life-threatening risks [25]. There are chances of a cure if treated promptly and properly [26]. LncRNAs have prospective clinical applications for the treatment of RB [7] with implications in the proliferation and apoptosis of tumor cells [6]. LncRNA HOTAIR might mark the abnormal regulation of the cell cycle [27]. This study demonstrated that lncRNA HOTAIR facilitated RB cell proliferation and repressed apoptosis via the miR-20b-5p/RRM2/PI3K/AKT axis.

HOTAIR propels carcinogenesis and shows an association with tumor initiation and progression, drug resistance, and poor survival rates of patients [28]. In previous studies concerning RB, most researchers have selected ARPE-19 cells as the control group [29–31]. The results of our study found no significant difference in HOTAIR expression between the two normal retinal cell lines ARPE-19 and RPE-1, and thus ARPE-19 cells could be utilized as the ideal control in our experiments. Similar to the aforementioned study, the current research showed that HOTAIR expression was elevated in RB cells. HOTAIR is implicated in the cell cycle process in glioblastoma [32] while silencing HOTAIR declines proliferation and promotes apoptosis of synoviocytes in osteoarthritis [33]. After delivering sh-HOTAIR into Y79 and HXO-RB44 cells with relatively high HOTAIR expression, we discovered that HOTAIR silencing repressed cell proliferation, enhanced apoptosis, and blocked the cell cycle progression of RB cells in the G0/G1 phase. Transplanting RB cells into the subcutaneous tissues of immunodeficient nude mice has been proved feasible in previous studies [34, 35]. In fact, intra-vitreal transplantation could better represent the clinical condition of RB and show practical significance due to the resemblance to the actual micro-environment of RB cells. However, due to the low success rates and high difficulties in the practical operation of intra-vitreal transplantation, direct observation, and detection of the tumor growth speed, we xenotransplanted RB cells into the subcutaneous tissues of the nude mice in the animal experiments, which confirmed the suppressive function of HOTAIR silencing on RB growth in vivo. Upregulation of HOTAIR promotes the progression and aggravation of RB, exhibiting its potential in acting as a diagnostic biomarker and therapeutic target for RB [12]. Downregulation of HOTAIR restrains the proliferation and invasion of RB cells and then inhibits RB progression by limiting the Notch signaling pathway [8]. Altogether, HOTAIR was upregulated in RB cells, and HOTAIR silencing weakened the RB cell proliferative abilities and enhanced apoptosis.

Generally, HOTAIR as a ceRNA modulates miRNA expression and affects the actions of miRNA on target genes, thus modifying tumor progression [12]. To get a better idea about the ceRNA mechanism of HOTAIR in RB cells, we predicted the downstream miRNAs of HOTAIR through multiple databases, among which miR-20b-5p was the focus of our study. The dysregulation of miR-20b-5p has been documented in various human malignancies, including endometrial cancer [36], colon cancer [16], and prostate cancer [14]. Likewise, we observed diminished expression of miR-20b-5p in the RB cell lines. Moreover, silencing HOTAIR upregulated the expression of miR-20b-5p. Our results corroborated that HOTAIR could target miR-20b-5p. Remarkably, downregulation of miR-20b-5p enhances RB cell proliferation and reduces apoptosis [17]. miR-20b-5p inhibits the cell cycle of colon cancer cells [16]. To testify the effect of miR-20b-5p downregulation on RB cells, Y79 and HXO-RB44 cells were treated with miR-20b-5p inhibitor. After downregulating miR-20b-5p, we observed facilitated cell cycle progression and proliferation and suppressed apoptosis of RB cells, evidenced by the alterations of proliferation- and apoptosis-association proteins. Furthermore, after the combined treatment of HOTAIR silencing and miR-20b-5p inhibition, the cell cycle progression and proliferation of RB cells were facilitated and apoptosis was suppressed. Similarly, we found that the combined treatment of HOTAIR silencing and miR-20b-5p inhibition amplified the expressions of Ki67 and Bcl-2 and decreased the expression of Cleaved Caspase-3, indicating that downregulation of miR-20b-5p partly abrogated the inhibitory function of silencing HOTAIR on RB. Then we sought to pinpoint the target gene regulated by lncRNA HOTAIR/miR-20b-5p. The target genes were predicted through various databases, and the coexpression relationship of genes was searched to further screen the target genes among candidates. Ultimately, two target genes with the highest scores (RRM2 and KPNA2) were obtained. Intriguingly, RRM2 plays a vital role in crucial cellular processes such as proliferation, invasion, migration, and senescence, *and* shows abundant expression in human malignancies and a close association with poor prognosis [19–21]. We observed increased levels of RRM2 in RB cells relative to those in normal retinal cells. It was confirmed that miR-20b-5p targeted RRM2. RRM2 plays a key role in cell cycle regulation and also acts as a potential factor during RB tumorigenesis [22]. RRM2 promotes the cell cycle progression in lung cancer [19]. After elevating RRM2 expression, RB cells manifested accelerated cell cycle progression, decreased number in the G0/G1 phase, increased number in the S phase, strengthened proliferation and inhibited apoptosis, proved by upregulation of Ki67 and Bcl-2 and downregulation of Cleaved Caspase-3. Functional rescue experiments were subsequently conducted to further investigate the role of RRM2 in RB cells, which confirmed that overexpression of RRM2 promoted the cell cycle progression and proliferation, and inhibited apoptosis of RB cells. RRM2 exhibits a strong correlation with Ki67 in adrenocortical cancer [37]. RRM2 upregulates the apoptosis-associated factor Bcl-2 and reduces Cleaved Caspase-3 in pancreatic cancer [38]. Our results elaborated that RRM2 overexpression upregulated Ki67 and Bcl-2 and downregulated Cleaved Caspase-3 in RB cells, illustrating that RRM2 overexpression inverted the protective effect of sh-HOTAIR on RB. A significant association can be noticed between RRM2 and the poor outcomes of RB patients [39]. In brief, HOTAIR upregulated RRM2 by competitively binding to miR-20b-5p, thereby accelerating RB cell proliferation and suppressing apoptosis.

Afterward, we dug deep into the downstream signaling pathway regulated by the ceRNA network of HOTAIR/miR-20b-5p/RRM2. HOTAIR accelerates colorectal cancer progression via the PI3K/AKT/mTOR pathway [40]. RRM2 regulates invasive and migrative abilities of breast cancer cells via the PI3K/AKT signaling pathway, thus inducing the metastatic potential of breast cancer [21]. As a common player in the aforementioned findings, PI3Ks are critical coordinators of intracellular signaling in response to the extracellular stimulation, and hyperactivation of the PI3K/AKT signaling cascades is not rare in human tumors [41]. Therefore, we detected the expressions of PI3K/AKT pathway-related proteins and found that HOTAIR silencing reduced the ratio of p-AKT/AKT and p-PI3K/PI3K, while RRM2 overexpression partially reduced the suppression of HOTAIR silencing on PI3K/AKT activation. Both in vivo and ex vivo assays unraveled the inhibitory functions of HOTAIR silencing on the phosphorylation levels of PI3K and AKT. Rb1 deletion and PI3K/AKT activation synergistically provoke RB in mice [42]. Repression of the PI3K/AKT contributes to retarding RB progression [43]. Altogether, HOTAIR silencing slowed down the tumor growth by inactivating the PI3K/AKT pathway.

## Conclusions

To sum up, HOTAIR upregulated RRM2 via sponging miR-20b-5p and then activated the PI3K/AKT pathway, thus facilitating RB cell proliferation and repressing apoptosis. This study was flawed, though. First of all, only two RB cell lines (Y79 and HXO-RB44) were studied. Secondly, only Y79 cells were used for transplantation in the xenograft study. Thirdly, only subcutaneous tissue transplantation was performed. Finally, this study lacked comprehensiveness due to the deficiency of clinical data and more cell lines. Additionally, the possibility of other miRNAs and target genes involved in the ceRNA network of HOTAIR remained unknown. In the future, more cell lines are to be used to perform comprehensive cell experiments and xenograft transplantation by injecting RB cells into the vitreous bodies of immunodeficient nude mice to preferably imitate the real micro-environment of RB tumors and look for other miRNAs and target genes engaged in the HOTAIR ceRNA network in RB, as well as other possible downstream signaling pathways.

## Data Availability

All the data generated or analyzed during this study are included in this published article.
